# Responses of eastern Chinese coastal salt marshes to sea-level rise combined with vegetative and sedimentary processes

**DOI:** 10.1038/srep28466

**Published:** 2016-06-23

**Authors:** Zhen-Ming Ge, Heng Wang, Hao-Bin Cao, Bin Zhao, Xiao Zhou, Heli Peltola, Li-Fang Cui, Xiu-Zhen Li, Li-Quan Zhang

**Affiliations:** 1 State Key Laboratory of Estuarine and Coastal Research, East China Normal University, Shanghai 200062, China; 2 School of Forest Sciences, University of Eastern Finland, Joensuu 80101, Finland; 3 Ministry of Education Key Laboratory for Biodiversity Science and Ecological Engineering, Fudan University, Shanghai 200433, China

## Abstract

The impacts of sea-level rise (SLR) on coastal ecosystems have attracted worldwide attention in relation to global change. In this study, the salt marsh model for the Yangtze Estuary (SMM-YE, developed in China) and the Sea Level Affecting Marshes Model (SLAMM, developed in the U.S.) were used to simulate the effects of SLR on the coastal salt marshes in eastern China. The changes in the dominant species in the plant community were also considered. Predictions based on the SLAMM indicated a trend of habitat degradation up to 2100; total salt marsh habitat area continued to decline (4–16%) based on the low-level scenario, with greater losses (6–25%) predicted under the high-level scenario. The SMM-YE showed that the salt marshes could be resilient to threats of SLR through the processes of accretion of mudflats, vegetation expansion and sediment trapping by plants. This model predicted that salt marsh areas increased (3–6%) under the low-level scenario. The decrease in the total habitat area with the SMM-YE under the high-level scenario was much lower than the SLAMM prediction. Nevertheless, SLR might negatively affect the salt marsh species that are not adapted to prolonged inundation. An adaptive strategy for responding to changes in sediment resources is necessary in the Yangtze Estuary.

Coastal salt marshes provide many ecological services as a habitat that supports biodiversity, carbon storage and coastal protection. However, coastal ecosystems tend to be vulnerable to global change, especially accelerated sea-level rise (SLR)^1^,^2^,^3^,^4^. The Fifth Assessment Report (AR5) by the Intergovernmental Panel on Climate Change (IPCC) predicted that the annual rate of global SLR by 2100 will exceed the rate observed during 1971–2010^5^. According to the latest bulletin of Chinese Sea Level released by the State Oceanic Administration^6^, the rate of SLR along China’s coastline is higher than the global average. Consequently, coastal salt marshes will be at great risk of degradation; the growth and survival of vegetation will be affected by an increased flooding duration under SLR^7^,^8^.

Many studies have predicted that coastal marshes will be submerged^3^,^9^,^10^, and some studies have attempted to assess the ability of coastal marshes to keep up with SLR by estimating sedimentary rates^2^,^11^. Among the approaches for studying the responses of coastal ecosystems to SLR, the Sea Level Affecting Marshes Model (SLAMM) has been frequently employed by managers for mitigation planning^1^,^12^,^13^. However, the SLAMM has not realistically modeled feedback of elevation in sediment dynamics or other critical biological processes^1^,^3^. Kirwan *et al*.^14^ also argued that marsh vulnerability tends to be overstated because assessment methods often fail to consider biophysical feedback processes known to accelerate soil building with SLR.

In the Yangtze Estuary, a large amount of sediment is deposited by the Yangtze River every year^15^. Sediment deposition in the estuary increases surface elevation and accretes at the seaward edge of the salt marshes, favoring the propagation and spread of vegetation^16^. Li and Yang^17^ investigated the trapping effect of marsh vegetation on suspended sediment. However, the extent to which the combined sedimentary and vegetative processes contribute to marsh resilience under SLR remains unclear. Recently, a grid-based salt marsh model for the Yangtze Estuary (SMM-YE) was developed to aid in the understanding of the biotic and abiotic factors that regulate the vegetation processes in the salt marshes^16^,^18^. The model accurately reproduced the spatiotemporal dynamics of the three dominant salt marsh species *Spartina alterniflora*, *Scirpus mariqueter* and *Phragmites australis* in the Yangtze Estuary.

In this study, both the SLAMM and the SMM-YE were used to simulate the effects of SLR in the Yangtze Estuary. Two national nature reserves in the Chongming Dongtan wetland and the Jiuduansha wetland were selected as representative subjects for study. The aims of this study are 1) to predict how salt marsh habitats in the Yangtze Estuary respond to the different SLR scenarios (the regional scenario and the IPCC scenario) during the 21^st^ century and 2) to assess the combined role of sedimentary accretion and vegetative processes in influencing the further vegetation structure and marsh resilience under SLR. In addition, mitigation measures to threats of SLR are briefly discussed.

## Materials and Methods

### Study area

**Figure 1:**
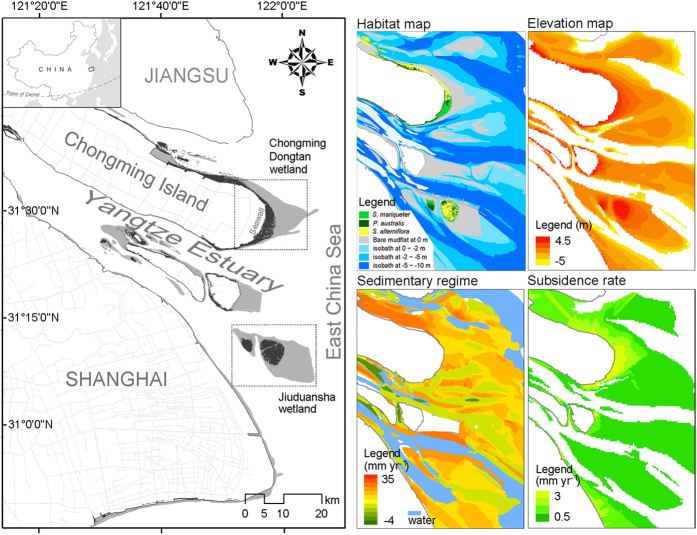
Location of the Chongming Dongtan (CDW) and Jiuduansha (JW) wetlands in the Yangtze Estuary with a classified map of salt marsh habitats (2008), current digital elevation map, sedimentary regime (2005–2010 investigation documents from the Survey Bureau of Hydrology and Water Resources of the Changjiang River Estuary, the Changjiang Water Resources Commission) and land subsidence. The maps were created from the authors’ data using ArcGIS 10.0 (www.esri.com/software/arcgis) and CorelDRAW Graphics Suite X6 (www.coreldraw.com/us/product/graphic-design-software).

The Yangtze Estuary is located on the eastern coast of China, which is neighbored by the Hangzhou Bay to the south and Jiangsu Province to the north and which opens to the East China Sea (Fig. 1). The Yangtze Estuary has an eastern Asian monsoon climate with an average annual temperature ranging from 15.2–15.8 °C. The average annual precipitation is approximately 1,022 mm, and the average humidity is approximately 82%^19^. Tidal movement in the estuary is irregular and semidiurnal, with maximum and average tide heights of 4.62–5.95 m and 1.96–3.08 m, respectively^15^.

There are two representative salt marshes in the estuary (Fig. 1). The Chongming Dongtan wetland (hereafter CDW, also the Chongming Dongtan Nature Reserve) is located on the eastern fringe of Chongming Island between 31°25′–31°38′N and 121°50′–122°05′E. The wetland covers an area of 242 km^2^ above 0 m isobaths based on the local Wusong bathymetric benchmark. The Jiuduansha wetland (hereafter JW, also the Jiuduansha Nature Reserve) is an isolated shoal between 31°03′–3°17′N and 121°46′–122°15′E. The wetland covers an area of 127 km^2^ above the 0 m isobaths. In the wetlands, tidal salt marshes with elevations less than 2 m are characterized by bare mudflats without any vascular plants. The mudflats between 2.0 and 2.5 m elevation mainly contain *S. mariqueter*. The coastal wetlands with elevations above 2.5–2.9 m are dominated by *P. australis* and *S. alterniflora*.

### SMM-YE

The detailed SMM-YE was constructed using the MATLAB^®^ (The MathWorks, Inc., Natick, MA, the U.S.) matrix platform^16^,^18^. The model consists of a spatial grid (1 m × 1 m) of interconnected cells, which are connected to all neighboring cells with a distribution probability for the spread of vegetation associated with each connection. Habitats of water, bare mudflat and dominant plant species (*P. australis*, *S. mariqueter* and *S. alterniflora*) are coded in each cell of the matrix using different numbers for identification. Each cell evolves according to the transition rules, which depend on the state of its neighboring cells.

**Figure 2:**
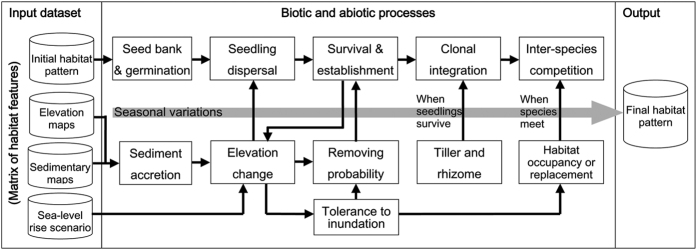
Schematic showing the modeling procedure and the related biotic and abiotic processes.

As the flow chart of the model (Fig. 2) indicates, the spatial and temporal patterns of the salt marsh are regulated by the interaction between biotic and abiotic processes, including the sedimentary regime, mudflat accretion, elevation change, seed bank deposition and germination, seedling dispersal, individual establishment and survival, tolerance to inundation stress, clonal integration and inter-specific competition. Moreover, the presence of marsh vegetation on a mudflat surface can trap the suspended sediment and increase the elevation, and the model includes the lateral accretion component found in these marshes. The detailed frame of the model with parameterization and validation is summarized in the Supplementary information.

The original parameters of the SMM-YE were determined under non-SLR conditions. To renew the key parameters of survival rate and seed-setting rate for SLR scenarios, we conducted a field experiment on the tidal flat to test the direct species-specific response to SLR (see the Supplementary information). The mesocosms of *S. alterniflora*, *S. mariqueter* and *P. australis* were grown at different flat elevations to simulate the inundation scenario of SLR. At CDW, 30 intact soil monoliths (16 cm diameter, 50 cm depth) consisting of vegetation seedlings were cored with PVC pipes for *S. alterniflora*, *S. mariqueter* and *P. australis*, with 10 replicates of each. The mesocosms were grown at three elevation levels of 3.0 m, 2.5 m and 2.2 m on the frontier tidal flat, and the surface elevation of the mesocosms was determined using a Real-Time Kinematic global positioning system (Ashtech, USA) with the local Wusong bathymetric benchmark. The information on the tidal regimes in the Yangtze Estuary referred to real-time monitoring as well as the official annals of the Shanghai Water Authority and the Shanghai Municipal Ocean Bureau.

Over the growing season, changes in the survival rate and seed-setting rate of plants were recorded. Through the relationship between plant survival and inundation duration (reflected by relative elevation), a simple elevation-based logistic function was used to explore the tipping point of the survival rate. We determined the irretrievable point to reflect the tolerance threshold (tipping point, *P*) of plant survival to the inundation duration with a hypothesis (*H*) of maximum-entropy.


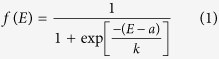



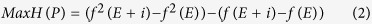


where *P* is identified when the maximum of the function is obtained, *E* is the mudflat elevation, *a* and *k* are the fitting parameters, and *i* is the regular sample unit of elevation.

### SLAMM

In this study, a parallel analysis was conducted using the SLAMM, a spatial model that simulates the dominant processes involved in salt marshes and shoreline modifications during periods of SLR^12^. The SLAMM simulates the primary processes of inundation, erosion, overwash, saturation, and accretion. The impacts of these processes on salt marshes are dependent on SLR. A flexible and complex decision tree, incorporating geometric and qualitative relationships, is used to represent transfers between land-cover categories. The software package and documentation for SLAMM version 6.0.1 (beta) are available at http://warrenpinnacle.com/prof/SLAMM/.

Vertical accretion is considered in SLAMM simulations using the spatial sedimentary regime and the average annual rate in the Yangtze Estuary. Accretion feedbacks based on wetland elevation relative to the tidal range were used in the simulations. The SLAMM uses a set of land-cover categories (codes) corresponding to each type of vegetation habitat. The categories defined for the CDW and JW were based on the classified salt marsh maps (see below). In addition, the model inputs included a digital elevation model, tidal data, rates of mudflat accretion, land subsidence, and various SLR scenarios.

### Initial data sources for modeling

#### Classified salt marsh maps

The classified salt marsh vegetation maps from 2008 for the CDW and JW were obtained through the national wetlands resource investigation and monitoring program (2012)^20^ (Fig. 1). The maps were opened in ArcGIS 10.0 and converted to land-cover polygons. For the SMM-YE, the MATLAB matrix for each habitat polygon was produced for projection^16^,^18^. For the SLAMM, the ASCII file of the salt marsh vegetation and other land properties with the various SLAMM codes were exported based on the translation information in the SLAMM 6.0.1 technical documentation^12^.

#### Digital elevation map

With the investigation program described above, the remotely sensed digital elevation data from 2008 for the CDW and JW were shared for use. The digital elevation map (DEM) of the study area was constructed using the Krasovsky 1940 Transverse Mercator coordinate system and the Transverse Mercator map projection. A single-cell DEM was 30 m × 30 m (Fig. 1). Nautical charts of the Yangtze Estuary from the Chinese Navy were used to provide isobath information, especially for the areas below sea level. The mean tidal level and isobaths of 0 m, −2 m and −5 m were delineated using ArcGIS 10.0.

#### Sedimentary regime

Long-term (1997–2010) monitoring data of the accretion/erosion rate (vertical change in elevation) were measured by the Survey Bureau of Hydrology and Water Resources of the Changjiang River Estuary (documented by the Changjiang Water Resources Commission)^21^. The data were obtained at the nearby Waigaoqiao tidal gauge station, which is close to Chongming Island, and are based on the local Wusong bathymetric benchmark (Fig. 1). The MATLAB matrix file of the sedimentary regime map was formatted as the inputs for the programs of the SMM-YE. To comply with the input requirements of SLAMM program, the ‘sub-sites’ of the sedimentary regime map with geographical property were partitioned following the technical menu^12^.

#### Land subsidence

Land subsidence in the coastal wetlands of the Yangtze Estuary occurs primarily in marine sediments (Fig. 1). The natural conditions include neotectonic movement and the natural settlement of poorly consolidated sediments^22^. Based on long-term (1980–2005) monitoring using laser ranging and global positioning system technology^23^, the average neotectonic subsidence rate is 1.5 mm year^−1^ in the Yangtze Estuary.

### SLR scenarios and simulations

In this study, the model simulations were based on two scenarios of regional and global projections adopted for the evaluation of SLR in the coastal salt marshes of CDW and JW, as the present SLR (PSLR) of 0.30 m (low level)^6^ and the IPCC RCP (Representative Concentration Pathway) 8.5 max scenario of 0.98 m (high level) by the end of 21^st^ century^5^. The simulation periods were evaluated using the combination of SLR and ecosystem processes in the short term (2008–2025), medium term (2025–2050) and long term (2050–2100) using the modeling procedures of the SMM-YE and the SLAMM. The same initial inputs were used in both models, i.e., the geographical data from the classified map of the salt marshes, digital elevation map, sedimentary regime, and land subsidence.

## Results

### Response of the salt marshes to SLR based on the SMM-YE

**Figure 3:**
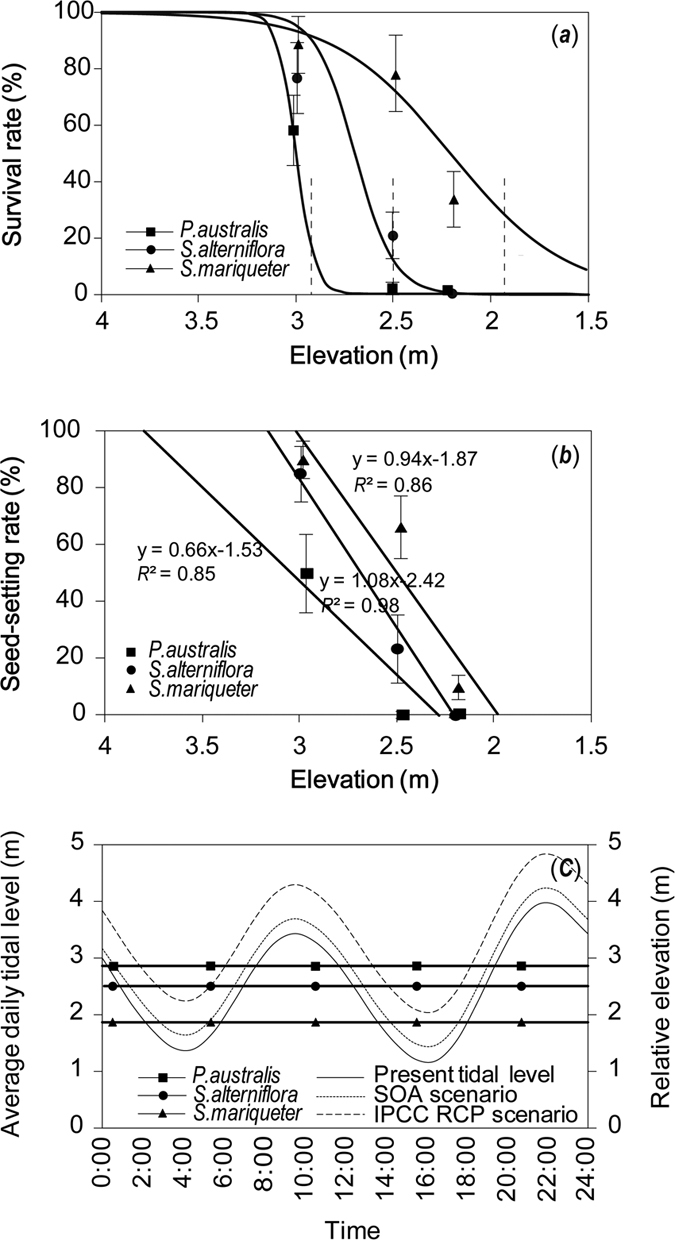
Responses of plant survival (**a**) and seed-setting rates (**b**) of the salt marsh species under inundation treatments (by variations of elevation). The dotted lines indicate the tipping points of plant survival under inundation stress. Daily tidal range and inundation duration for the different species (**c**) under the non-SLR and SLR scenarios are shown.

**Figure 4:**
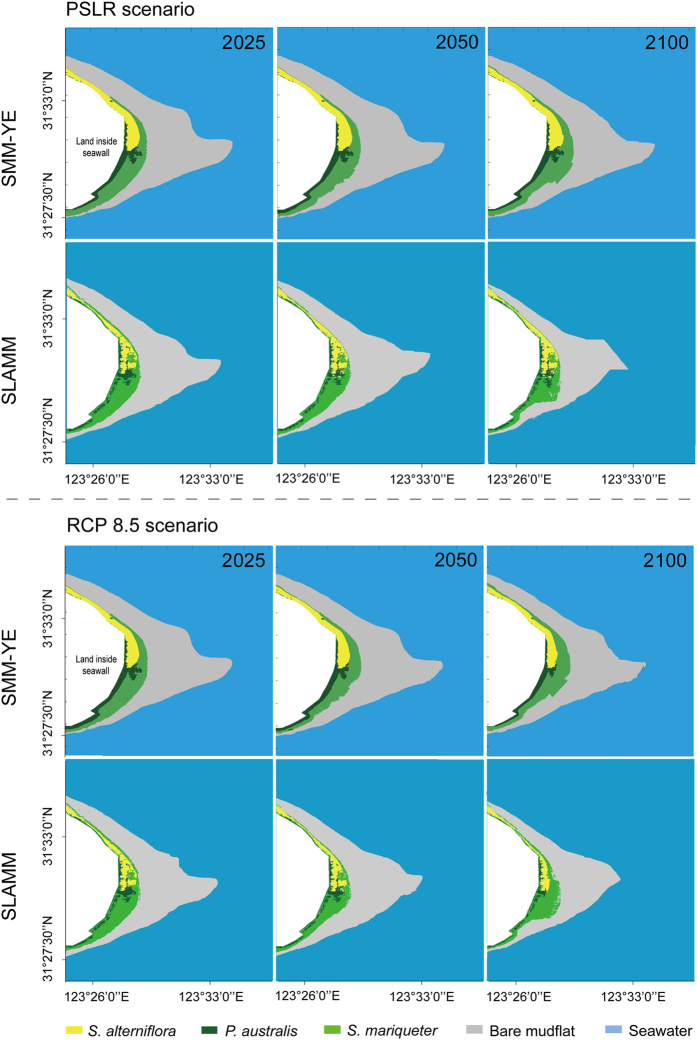
Spatial projections of the salt marsh (above 0 m isobaths) in the Chongming Dongtan wetland (CDW) over the short term (2025), medium term (2050) and long term (2100) based on the SMM-YE and SLAMM under the PSLR scenarios. The maps were created from the authors’ data using MATLAB R2010b (se.mathworks.com/products).

**Figure 5:**
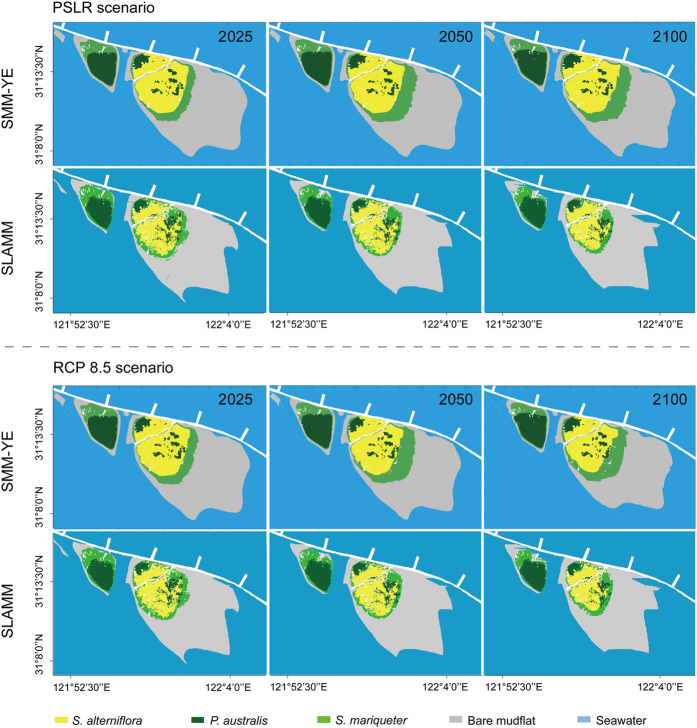
Spatial projections of the salt marsh (above 0 m isobaths) at the Jiuduansha wetland (JW) over the short term (2025), medium term (2050) and long term (2100) based on the SMM-YE and SLAMM under the PSLR scenarios. The maps were created from the authors’ data using MATLAB R2010b (se.mathworks.com/products).

The field experiment revealed the responses of salt marsh vegetation to varying inundation conditions in terms of flat elevation (Fig. 3a). The down-regulated logistic curve provided the tipping points of plant survival as 2.5 m for *S. alterniflora*, 1.9 m for *S. mariqueter* and 2.9 m for *P. australis*. The experiment also showed that the seed-setting rates decreased (linear regression, *R*^2 ^= 0.85–0.98) with the reduction in elevation for all three species (Fig. 3b). Based on the mean daily tidal range, the daily inundation duration for *S. alterniflora*, *S. mariqueter* and *P. australis* was estimated to be approximately 10, 13 and 8 h day^−1^, respectively (Fig. 3c). With the key vegetative parameters of different salt marsh species in relation to SLR, SMM-YE simulations predicted the response of the salt marsh habitats above 0 m isobaths at CDW (Fig. 4) and JW (Fig. 5) to the PSLR and RCP 8.5 scenarios.

The SMM-YE also accounted for the combination of sedimentary and vegetative processes, which result in the accretion of mudflats and the lateral expansion of vegetation. During the simulation period of 2008–2025, the habitat area of *P. australis*, *S. alterniflora* and *S. mariqueter* at CDW and JW increased by 2–20% and 8–22%, respectively, regardless of the SLR scenarios (Table 1). This increase was attributed to higher rates of mudflat accretion and spatial spread seaward than the rate of submergence (Figs 4 and 5). Because of the vegetation colonization and SLR, the bare mudflat area decreased by 4% and 10% at CDW and by 6% and 7% at JW under the PSLR and RCP 8.5 scenarios, respectively. By 2025, the total salt marsh area at CDW and JW increased by 6% under the PSLR scenario, while 2% was at risk of submersion under the RCP 8.5 scenario (Table 1).

During the 2008–2050 simulation period, the SMM-YE predicted an increase in area for *P. australis* and *S. alterniflora* due to lateral expansion seaward at both wetlands, regardless of the SLR scenarios (Table 2). The area increase in *S. mariqueter* was much greater (by 21% and 30% at CDW and 38% and 42% at JW under the PSLR and RCP 8.5 scenarios, respectively) than that of *P. australis* and *S. alterniflora*. The areas of bare mudflat declined by 6% and 16% at CDW and by 13% and 15% at JW under the PSLR and RCP 8.5 scenarios, respectively. By 2050, the total habitat area at CDW and JW increased by 4% under the PSLR scenario, while the RCP 8.5 scenario resulted in a 5% decline (Table 2).

By the end of the simulation period (2100), the area of *S. alterniflora* increased by 12% at CDW and 14% at JW under the PSLR scenario but decreased by 7% at CDW and 8% at JW under the RCP 8.5 scenario (Table 3). The increase in the area of *S. mariqueter* was much greater (by 40% and 32% at CDW and 23% and 21% at JW under PSLR and 32% under RCP 8.5, respectively) than that of *P. australis*. This increase was attributed to the combined effects of spatial spread and conversion from other vegetation under stress of submergence (Figs 4 and 5). The area of bare mudflat decreased by 12% and 25% at CDW and 15% and 21% at JW under the PSLR and RCP 8.5 scenarios, respectively. By 2100, the total area of the salt marsh habitats at CDW and JW under the PSLR scenario increased by 3%, while under the RCP 8.5 scenario, a loss of 9% was observed (Table 3).

### Response of the salt marshes to SLR based on the SLAMM

The SLAMM did not account for the lateral accretion of mudflat and expansion of vegetation with the annual sedimentary rates. As presented in Figs 4 and 5, the changes in area of the marsh vegetation were not obvious during the 2008–2025 simulation period. The SLAMM predicted that SLR would lead to a decline in salt marsh area by 4% and 8% at CDW and 4% and 5% at JW under the PSLR and RCP 8.5 scenarios, respectively, during 2008–2025 (Table 1).

During the simulation period of 2008–2050, the habitat of the *S. alterniflora* seaward area was converted to *S. mariqueter*, and the bare mudflats at both wetlands (Figs 4 and 5) showed a reduction in area (Table 2). The area of *S. mariqueter* and *P. australis* also decreased, regardless of the SLR scenarios. By 2050, 10% and 14% of the total habitat area at CDW and JW were converted to open water under the PSLR and RCP 8.5 scenarios, respectively.

By 2100, *S. mariqueter* and *S. alterniflora* in the frontier marsh were converted to bare mudflats at both wetlands. By 2100, 18% and 29% of the total habitat area at CDW and 15% and 22% at JW was submerged under the PSLR and RCP 8.5 scenarios, respectively (Table 3).

### Role of combination of sedimentary and vegetative processes

**Figure 6:**
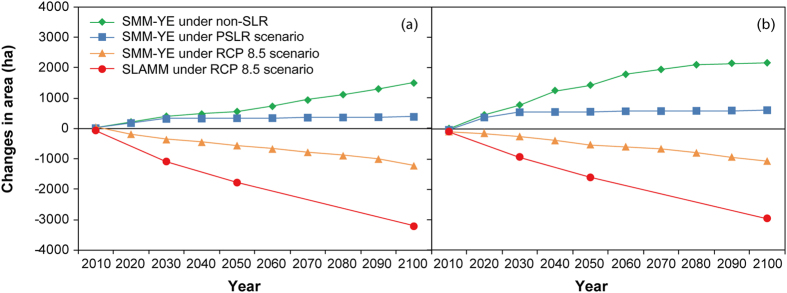
Changes in the total salt marsh habitat area at the Chongming Dongtan wetland (CDW, (**a)**) and the Jiuduansha wetland (JW, (**b)**) over the simulation period of 2008–2100 based on the SMM-YE and SLAMM simulations and the scenarios of non-SLR, PSLR and RCP 8.5.

Predicted changes in habitat area of the salt marshes from the SMM-YE and the SLAMM were compared to identify the combined effects of sedimentary accretion and vegetative processes (Fig. 6). When the scenario of SLR was not applied (non-SLR), the SMM-YE predicted that the total salt marsh area above 0 m isobaths increased by around 1500 ha at CDW and 2200 ha at JW by 2100. However, the SLAMM could not simulate the lateral mudflat accretion and spatial spread. When the PSLR scenario was assumed, the SMM-YE predicted a small increase in the area of salt marshes by 2100. The RCP 8.5 scenario led to a decrease in the area of salt marshes based on the SMM-YE, while the SLAMM predicted a much greater loss during the simulation period.

## Discussion

### Features of the salt marshes in the Yangtze Estuary under SLR

The goal of this study was to understand the potential effects of SLR on the salt marshes in the Yangtze Estuary. The SLAMM predicted net habitat loss of 4–29% at CDW and 4–22% at JW during different simulations. Salt marshes suffered greater losses under high SLR (RCP 8.5) than under the low scenario (PSLR). In the coastal salt marshes, the vegetation along the seaward edge of the marsh will degrade to be dominated by pioneer species or unvegetated land because of long submergence at the lower elevations^1^,^3^. Platform deepening may cause a marsh to quickly submerge below elevations under high rates of SLR, representing an important threshold for vegetation growth at which the marsh platform submerges or erodes because of subtidal elevations^2^,^7^.

However, the locally developed SMM-YE for the Yangtze Estuary accounted for the processes of lateral mudflat accretion, vegetation dynamics and sediment trapping by vegetation and indicated resilience of the salt marshes to SLR. Under low level of SLR, total salt marsh areas at both CDW and JW increased by relatively small increments, as indicated by the long-term simulation (i.e., 2008–2100). This minimal effect occurs because the deposition of river sediment in the estuary and along the coast functions as a form of self-regulation in response to SLR^3^,^10^. When SLR scenarios were excluded, the current sedimentary rate in the Yangtze Estuary will increase the areas of salt marshes above 0 m isobaths at CDW and JW at the end of the 21^st^ century, as simulated by the SMM-YE. Under the maximum scenario of RCP 8.5, the decrease in the habitat area predicted by the SMM-YE is much lower than the output from the SLAMM over the corresponding years.

The resilience to SLR might also be attributed to the vegetation process module in the SMM-YE, which assumes that seeds and seedlings will spread to neighboring areas via tidal currents and establish new meadows. The model also considers the ability of salt marsh vegetation to trap suspended sediment, exhibiting a positive feedback loop between vegetation expansion and sediment deposition^7^,^24^,^25^. For instance, *S. alterniflora* can attenuate hydrological energy significantly and enhance sediment accretion, resulting in higher seedling recruitment and a wider range of expansion^26^. Based on a field measurement at CDW, the introduced *S. alterniflora* is more efficient in trapping suspended sediment than the native species^17^. The development of vegetative tillering and growth of rhizomes underground further benefits the spatial spread of vegetation in the salt marshes^27^.

Furthermore, our model is able to simulate the spatiotemporal dynamics of the plant community in the salt marsh, including the two native dominant species *P. australis* and *S. mariqueter* and the exotic species *S. alterniflora*. In addition to the vegetation establishment for the individual species, the inter-species relationship was parameterized in the SMM-YE, showing a close correlation between the simulation and observation data of community dynamics (based on remote sensing data)^16^. In the Yangtze Estuary, *S. mariqueter* that grows on the mudflats with low elevation thresholds is the pioneer vegetation^28^. Previous studies demonstrated that *S. alterniflora* quickly invaded both unvegetated zones and *S. mariqueter* habitat in the estuary since the introduction of *S. alterniflora*^29^,^30^,^31^, which was also reproduced by our model^16^,^18^.

Under the PSLR scenario, the area of both *S. mariqueter* and *S. alterniflora* increased in the frontier of the marsh flat because sedimentary accretion led to spatial spread. However, the RCP 8.5 scenario reduced the area of the exotic species and resulted in the recolonization of *S. mariqueter* in the low-lying habitats during the later simulation period. The range expansion of both native and exotic salt marsh species relied heavily on the availability of habitat and the accretion rate of intertidal mudflats^16^. A fixed-point field monitoring study showed that a regime of accretion benefited from the expansion of *S. alterniflora* seaward and its invasion into the *S. mariqueter* zone in the advancing front^32^, while the invasion of *S. alterniflora* was significantly weak and the original pioneer species *S. mariqueter* remained under a relatively stable regime. These responses indicated that the current sedimentary rate for elevation accretion could not support the rapid rate of habitat colonization by *S. alterniflora* that occurred in the high-elevation habitat during the past decade. In the current study, the field experiment on species-specific tolerance to waterlogging also revealed the greater tolerance of *S. mariqueter* to prolonged inundation relative to *S. alterniflora* and *P. australis* in the frontier of marsh flats. Recently, a revegetation practice for the native species at CDW indicated that *S. mariqueter* could survive and colonize in the mudflats with very low elevation^33^. Therefore, the area of *S. mariqueter* will increase due to reduced stress from *S. alterniflora*, potentially leading to recolonization in the invaded habitat by *S. alterniflora* under a high-SLR scenario. Although the test showed the lowest tolerance of *P. australis* to inundation, its area was not decreased under SLR because the distribution zone of *P. australis* is located in high-elevation habitat, which is subjected to less flooding stress.

Nevertheless, another potential risk of prolonged flooding under SLR probably results in a significant reduction of seed-setting rates (based on the mesocosm experiment) of all species. This reduction might negatively affect the colonization of vegetation generation by generation.

### Uncertainty analysis

The SMM-YE did not simulate changes in marsh accretion but substituted historical average sedimentary rates from long-term records (1997–2010). The deposition process is based on the concentration of suspended sediment and organic material deposited during each period of tidal inundation^34^. The sediment deposition rates in the marsh are largely controlled by the duration and frequency of tidal inundation^2^,^25^. Therefore, potential changes in sediment loading and tidal flooding with SLR may lead to uncertainty in the marsh accretion estimates.

The core of the model is the inundation tolerance of different plant species to submergence. The sensitivity analysis in terms of threshold of plant survival under inundation (by plus or minus 15%) showed that the model results were sensitive to changes in the species-specific tolerance thresholds to submergence. This study used a field experiment with three inundation levels to estimate the tipping points of survival under waterlogging, probably leading to an underestimation. Moreover, the model was calibrated based on previous field observations and empirical assumptions, ignoring the plasticity of coastal vegetation. Pan *et al*.^35^ demonstrated that wetland plants can display morphological plasticity in response to inundation. Morris *et al*.^2^ also reported that increasing the inundation of salt marshes may increase macrophyte production and lead to increased vertical accretion. In this respect, the improvement of field trial and the model updating are needed.

### Management implications

The SMM-YE in this study showed that salt marshes could be resilient to SLR threats through the lateral accretion of mudflats, vegetation propagation and sediment trapping by vegetation. However, China’s coastal wetlands have been enclosed by thousands of kilometers of seawalls because of planning for economic growth, especially in East China^36^,^37^. The migration of salt marshes landward is not possible as a result of the restrictions caused by “coastal squeeze.” Additionally, the large amount of hydraulic engineering on the Yangtze River upstream has decreased the sediment discharge, leading to a continuous reduction in the sedimentation rate in the Yangtze Estuary^38^. The insufficient sediment supply would exacerbate the risk of SLR for coastal marshes^9^.

In addition, a nine-year nutrient enrichment experiment showed that anthropogenic eutrophication might rapidly result in marsh erosion and loss around the coastal landscape^39^. Eutrophication has become increasingly serious in the Yangtze Estuary and the adjacent East China Sea because of human activities^40^. This process will consequently reduce the capacity of coastal regions to adapt to expected SLR.

Therefore, management of sediment resource and nutrient loading in estuaries and coastal zones is a crucial mitigation strategy because the increased availability of sediment allows the coastal wetlands to keep pace with SLR. The Yangtze Estuary is an important navigation channel, and more than 70 Mt of sediment is dredged each year to maintain the deep-water channel^41^. Two effective soft management options available to mitigate the impacts of SLR are the use of dredged sediment to encourage mudflat or salt marsh development and the reestablishment of salt marsh pioneer vegetation to promote sedimentation by slowing the flow and reducing wave energy.

## Additional Information

**How to cite this article**: Ge, Z.-M. *et al*. Responses of eastern Chinese coastal salt marshes to sea-level rise combined with vegetative and sedimentary processes. *Sci. Rep.* **6**, 28466; doi: 10.1038/srep28466 (2016).

## Supplementary Material

Supplementary Information

## Figures and Tables

**Table 1:** Predicted change percentage (%) of salt marsh habitats (above isobaths of 0 m) at CDW and JW over the short term (2008–2025) under the scenarios of PSLR and RCP 8.5 with the SMM-YE and the SLAMM.

Habitat	CDW				JW				Total study area			
	PSLR		RCP 8.5		PSLR		RCP 8.5		PSLR		RCP 8.5	
	SMM-YE	SLAMM	SMM-YE	SLAMM	SMM-YE	SLAMM	SMM-YE	SLAMM	SMM-YE	SLAMM	SMM-YE	SLAMM
2008–2025												
*S. mariqueter*	(+) 12	(−) 0	(+) 20	(−) 3	(+) 17	(+) 3	(+) 22	(+) 5	(+) 14	(+) 1	(+) 21	(+) 0
*P. australis*	(+) 2	(+) 0	(+) 2	(+) 0	(+) 11	(+) 0	(+) 8	(+) 0	(+) 8	(+) 0	(+) 6	(+) 0
*S. alterniflora*	(+) 8	(−) 5	(+) 5	(−) 17	(+) 13	(−) 1	(+) 11	(−) 4	(+) 11	(−) 4	(+) 9	(−) 8
Bare mudflat	(−) 4	(+) 5	(−) 10	(+) 2	(−) 6	(−) 7	(−) 7	(−) 8	(−) 5	(−) 1	(−) 9	(−) 3
Total	(+) 4	(−) 4	(−) 3	(−) 8	(+) 6	(−) 4	(−) 1	(−) 5	(+) 6	(−) 4	(−) 2	(−) 6

CDW: Chongming Dongtan wetland; JW: Jiuduansha wetland.

**Table 2:** Predicted change percentage (%) of salt marsh habitats (above 0 m isobaths) at CDW and JW over the medium term (2008–2050) under the scenarios of PSLR and RCP 8.5 with the SMM-YE and the SLAMM.

Habitat	CDW				JW				Total study area			
	PSLR		RCP 8.5		PSLR		RCP 8.5		PSLR		RCP 8.5	
	SMM-YE	SLAMM	SMM-YE	SLAMM	SMM-YE	SLAMM	SMM-YE	SLAMM	SMM-YE	SLAMM	SMM-YE	SLAMM
2008–2050												
*S. mariqueter*	(+) 21	(−) 18	(+) 30	(−) 25	(+) 38	(−) 12	(+) 42	(−) 15	(+) 28	(−) 16	(+) 35	(−) 21
*P. australis*	(+) 10	(+) 0	(+) 3	(−) 3	(+) 14	(−) 1	(+) 9	(−) 1	(+) 13	(−) 1	(+) 7	(−) 2
*S. alterniflora*	(+) 16	(−) 15	(+) 5	(−) 20	(+) 21	(−) 3	(+) 10	(−) 9	(+) 19	(−) 7	(+) 8	(−) 13
Bare mudflat	(−) 6	(+) 2	(−) 16	(−) 2	(−) 13	(−) 10	(−) 15	(−) 13	(−) 10	(−) 4	(−) 16	(−) 8
Total	(+) 3	(−) 11	(−) 5	(−) 16	(+) 4	(−) 10	(−) 4	(−) 12	(+) 4	(−) 10	(−) 5	(−) 14

CDW: Chongming Dongtan wetland; JW: Jiuduansha wetland.

**Table 3:** Predicted change percentage (%) of salt marsh habitats (above 0 m isobaths) at CDW and JW over the long term (2008–2100) under the scenarios of PSLR and RCP 8.5 with the SMM-YE and the SLAMM.

Habitat	CDW				JW				Total study area			
	PSLR		RCP 8.5		PSLR		RCP 8.5		PSLR		RCP 8.5	
	SMM-YE	SLAMM	SMM-YE	SLAMM	SMM-YE	SLAMM	SMM-YE	SLAMM	SMM-YE	SLAMM	SMM-YE	SLAMM
2008–2100												
*S. mariqueter*	(+) 40	(−) 32	(+) 32	(−) 39	(+) 23	(−) 44	(+) 21	(−) 47	(+) 33	(−) 37	(+) 28	(−) 42
*P. australis*	(+) 11	(−) 2	(+) 8	(−) 23	(+) 12	(−) 2	(+) 6	(−) 3	(+) 12	(−) 2	(+) 7	(−) 11
*S. alterniflora*	(+) 12	(−) 14	(−) 7	(−) 22	(+) 14	(−) 6	(−) 8	(−) 29	(+) 13	(−) 9	(−) 8	(−) 27
Bare mudflat	(−) 12	(−) 53	(−) 25	(−) 60	(−) 15	(−) 14	(−) 21	(−) 19	(−) 14	(−) 34	(−) 23	(−) 40
Total	(+) 2	(−) 18	(−) 11	(−) 29	(+) 3	(−) 15	(−) 8	(−) 22	(+) 3	(−) 16	(−) 9	(−) 25

CDW: Chongming Dongtan wetland; JW: Jiuduansha wetland.
