# Supplementary material for: Responses of eastern Chinese coastal salt marshes to sea-level rise combined with vegetative and sedimentary processes
Source: Sci Rep. 2016 Jun 23;6:28466. doi: 10.1038/srep28466 (PMC4917823; doi:10.1038/srep28466)
Supplement: Supplementary Information [file srep28466-s1.doc]

**Supplementary information**

**Responses of eastern Chinese coastal salt marshes to sea-level rise combined with vegetative and sedimentary processes**

Zhen-Ming Ge*^,1,2^, Heng Wang^1^, Hao-Bin Cao^1^, Bin Zhao^3^, Xiao Zhou^2^, Heli Peltola^2^, Li-Fang Cui^1^, Xiu-Zhen Li^1^, Li-Quan Zhang^1^

1. *State Key Laboratory of Estuarine and Coastal Research, East China Normal University, 200062 Shanghai, China*
2. *School of Forest Sciences, University of Eastern Finland, 80101 Joensuu, Finland*

*3. Ministry of Education Key Laboratory for Biodiversity Science and Ecological Engineering, Fudan University, Shanghai 200433, China*

* **Corresponding author**

*E-mail address*: [zmge@sklec.ecnu.edu.cn](mailto:zmge@sklec.ecnu.edu.cn)

Address: Hehai Building, East China Normal University, Road North Zhongshan, 200062 Shanghai, China

Supporting information includes the field experiment design and detailed frame of the SMM-YE model with parameterization and validation.

**Field experiment design**

**
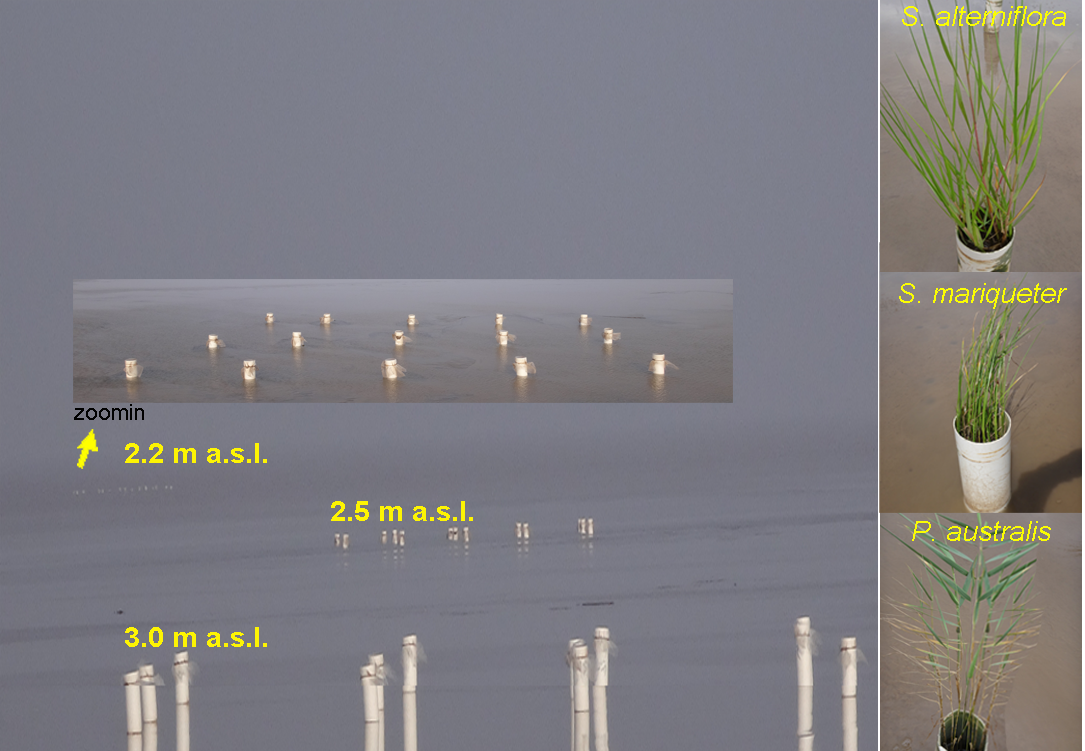
**

**Figure S1**. Field experiment design on responses of plant survival and seed-setting under inundation stress with mesocosms of the dominant salt marsh species *S. alterniflora*, *S. mariqueter*, and *P. australis*. The photographs were taken by Ge Z.M. at the Chongming Dongtan wetland in the Yangtze Estuary.

**SMM-YE model with validation**

***The sedimentary regime in relation to mudflat elevation changes***

The simulation of vegetation expansion and establishment depends on mudflat accretion. The model simulated mudflat accretion by determining the amount of sediment deposited and calculating the increase in elevation during each period of one year, based on the seasonal variations (flood and dry seasons) observed on monitoring strips established previously at CDW ^1^. In order to estimate spatially the vertical elevation changes at each cell, the model divides the geographic matrix (*S*) with the annual mean sedimentary rate into 12 month-specific components (*S*_i_, i = 1–12 months) to derive the mudflat elevation (*E*) on the elevation matrix and the subsequent increase in lateral mudflat area (when *E* is above 0 m) of CDW and JW on the habitat matrix at each time-step (*t*).

$\sum_{i=1}^{n=12} S_{i}=S$ (1)

$E(t+1)=E(t)+S_{i}$ (2)

***The growing period and simulation of reproduction***

In the model the period March–October was defined as the growing season, including the processes of seed germination, seedling dispersal and establishment, vegetative growth and clonal integration and seed bank deposition within the salt marsh vegetation ^2^. The residual months (November–February) corresponded to the inactive period for plants.

Measurements for the seasonal seed bank dynamics and germination rate recorded from March to October within the growing season during 2007–2010, were used to drive the model. Although the highest density of seeds in the soil seed bank were recorded in autumn and winter ^3^, most of seeds (c.96–98%) are lost before the next growing season, due to fungal attack, decay and tidal overwash ^1,3^. Furthermore, low temperature during winter season limited germination for the seeds.

***Dispersal and establishment***

For *P. australis* and *S. alterniﬂora*, the model divided the growing season of vegetation into two periods of expansion, including a period of ‘long-distance’ seedling dispersal by tidal currents (through growing season) followed by a period of vegetative growth and clonal integration (May–October). The pioneer native plant *S. mariqueter* was assumed to spread mainly through its corms and rhizomes, since few seeds are found in the seed banks on the bare mudflats ^2^.

In the matrix of seedling dispersal, cell values are considered as occupied (1) by the seedlings or not (0) and are dispersed from meadows (occupied cells) to the bare mudflats (cells without plants) in the corresponding matrix. The number of living seedlings (*N*) produced for dispersal was regarded as equal to the seedlings that germinated in each month during a growing season and was determined by a sum function of vegetation distribution area on salt marsh (*A*), density of seed bank (*D*_seed_) and germination rate (*R*_g_) for each species in the corresponding row (month).

$N=\sum_{A} (D_{\text{seed}}\times R_{\text{g}})$ (3)

The seedlings were distributed via a random allocation process and allowed for multiple seedlings of each species in each cell.

During the growing season, the model assumed that the seedlings of *P. australis* and *S. alterniﬂora* could be transported in any of eight directions by the tidal currents as a stochastic process. The seedling amount distributed from the original cells (*V*_i.j_) within eight directions around each cell was based on a separable space-time Poisson distribution for determining number of dispersal events in the region of each direction:

$\lambda(x,t)=f(x)\lambda(t)$ (4)

(5)

$\int_{V} f(x)dx=N$ (6)

where *λ* is the arrival rate, *t* is the time step, *x* is a random direction, *V* is the spatial matrix for seedling dispersal and *f*(*x*) represents the spatial probability density function of these stochastic events.

The seedling dispersal event was assigned a dispersal distance derived from the previous field measurements for the different species. The mean transportation distance of seedlings from the vegetation meadow over one growing season was 60–100 m for *S. alterniﬂora* and around 10 m for *P. australis* ^1,4−6^.

Once seedlings become established, they form tussocks quickly by vegetative tillering and growth of rhizomes, finally merging into extensive meadows. The clonal integration by tillers and rhizomes was modeled as a neighboring spreading of plant cells, based on the expansion mode of “Moore neighborhood” comprising the eight cells surrounding a central cell. The growth and horizontal spread of rhizomes can reach around 1 m per month during the period May–September ^5^. Accordingly, the monthly (one time step) rate of spread was incorporated into the model by defining the radius of the “Moore neighborhood” as 1 for the salt marsh vegetation.

***Environmental stress and tolerance***

The probability of survival of seedlings surviving on a patch depends on the suitability of the habitat and the environmental stress. Empirically, the establishment probability of seedlings was dependent on the hydrodynamic conditions of the mudflat front, i.e. the closer patch is to the vegetation meadow with a higher elevation and weak tidal flow intensity, the higher the number and density of seedlings established. On the contrary, the nearer a patch is to the sea with a lower elevation and stronger tidal flow intensity, the fewer the number and density of seedlings established ^3,5,7^. The probability (*P*) of seedling establishment is estimated by an asymptotic diminishing function of dispersal distance (*d*, distance from meadow front seaward to dispersal boundary).

$P(0\leq x\leq d)=\int_{0}^{d} f(x)dx$ (7)

Inundation duration is also a significant stress for salt marsh plants. Once again, from previous field surveys and measurements, differences were found in the tolerance of seedlings or adults to submergence ^2,4,8^, based on the mean duration of daily tidal inundation in relation to mudflat elevation above the local Wushong bathymetric benchmark.

In order to set the spreading and survival limits for the seedlings of salt mash plants in the model, the mudflat elevation of the study area was assumed to reflect the mean daily inundation duration. The elevation matrix map was then overlain on top of the matrix of plant distribution to determine the habitable cells for plant survival and establishment. A discriminant function was employed to assign a value of 1 (true) or 0 (false) to decide whether the grid could be occupied by each of the salt mash plants, taking the elevation (*E*) into account.

$f(x)=\left\{\begin{matrix} 1, & 4.2\geq E\geq2.9 & \text{P. australis and } \\ 0, & E<2.9 & \\ & & \\ 1, & 4.2\geq E\geq2.5 & \text{S. alterniflora} \\ 0, & E<2.5 & \\ & & \\ 1, & 4.2\geq E\geq1.9 & \text{S. mariqueter} \\ 0, & E<1.9 & \end{matrix}\right\}$ (8)

***Interspecific competition***

Chen et al. ^9^ reported that *S. alterniflora* was a strong competitor with *S. mariqueter*. The introduction of *S. alterniflora* to a *S. mariqueter*-dominated community resulted in a significant decrease in the abundance, coverage, seed production and fresh corm output of *S. mariqueter* during the growing season. Data obtained through remote sensing also showed that *S. mariqueter* was almost completely replaced by *S. alterniflora* at the monitoring sites in the Yangtze Estuary ^4,6^. In contrast, *P. australis* and *S. alterniflora* generally showed non-aggression to each other and share similar niches in the intertidal zones ^10^.

***Sedimentation enhanced by marsh vegetation***

The presence of marsh vegetation on a mudflat surface can trap the suspended sediment and increase the elevation ^11^. Li and Yang ^12^ quantified the abilities of the three dominant species in the Yangtze Estuary to trap suspended sediment in the different salt marsh habitats. The model was designed to include the vertical accretion component found in these marshes. Based on the plant species, plant sediment trapping and dry bulk density of sediment measured in the Yangtze Estuary, we calculated the value of *E*_v_:

$E_{\text{v}}=\frac{S_{\text{T}}}{D_{\text{s}}}k$ (9)

where *S*_T_ is the amount of sediment trapped annually by the different plant species (see ^12^ for the data), *D*_s_ is the dry bulk density of the sediment, and *k* is a species-specific adjusting coefficient (10% for *P. australis* and *S. alterniflora*, 5% for *S. mariqueter*).

***Data sources for model calibration***

A number of other research studies conducted on the salt marshes of the Yangtze Estuary lie behind the parameterization and calibration of the current version of the model and further details are presented in Table S1. The model parameters (or running rules) have either been measured or estimated in previous research performed in relation to the growing periods and reproduction strategies of the species, their seed banks and germination characteristics, seed/seedling dispersal, seedling survival and establishment, clonal integration and expansion range, environmental stress, tolerance and interspecific competition.

**Table S1.** Sources of research data used for the parameterization and calibration of the model.

| Item | Description | Value | Deviation | Source |
| --- | --- | --- | --- | --- |
| Elevation change | Sedimentation rate-induced elevation change on mudflat habitats | see Fig. 1 | / | 13 |
| Seed bank and germination | Seasonal seed bank and germination variations for the salt marsh vegetation | / | ±5% | ^3,5,14^;  This study (see Fig. 3) |
| Seed/seedling dispersal | Recorded maximum distance of seedling dispersal with tide at the front of mudflat seaward | 80 m for *S. alterniﬂora*  8 m for *P. australis* | ±20 m  ±2 m | 1−3,5,6 |
| Seedling survival and establishment | Seedling survival as a function of distance between seedlings and vegetation meadow at the marsh edge | 0–100% based on Eq. 6 | ±5% |
| Clonal integration and expansion range | Lateral reproduction rate of salt marsh vegetation by tillering and rhizoming | 1 (m) | / |
| Environmental stress and tolerance | Threshold of inundation duration (converted to elevation) determining the establishment of salt marsh vegetation | 2.9 m for *P. australis*  2.5 m for *S. alterniﬂora*  1.9 m for *S. mariqueter* | ±5% | This study |
| Interspecific competition | Competitive balance between introduced and native salt marsh plants | *S. alterniﬂora* > *S. mariqueter*  *P. australis* > *S. mariqueter*  *S. alterniﬂora* = *P. australis* | / | 4,8−10 |

***Validation of the dynamic salt marsh model***

Local validation of the model accuracy was performed for the three dominant salt marsh species at both CDW (easternmost part of the habitat) and JW. The documented spatiotemporal dynamics of the salt marsh vegetation for 2000, 2004, 2006, 2008 ^4,6,8^ and 2011 (unpublished data) were used to evaluate the performance of the model. The simulated vegetation distribution in the salt marshes showed a similar pattern to the observed dynamics at both CDW and JW during the corresponding years, and the accuracy of the distribution of each salt marsh species was acceptable (Fig. S2). The reasonable outputs of the model were attributed to careful parameterization based on previous empirical field measurements of both biotic and abiotic processes in the salt marsh ^15^. Seedling dispersal and establishment on mudflat plays an essential role in the expansion of the range of the exotic species *S. alterniflora* and the long-term impact on the plant community dynamics in the Yangtze Estuary ^16^.


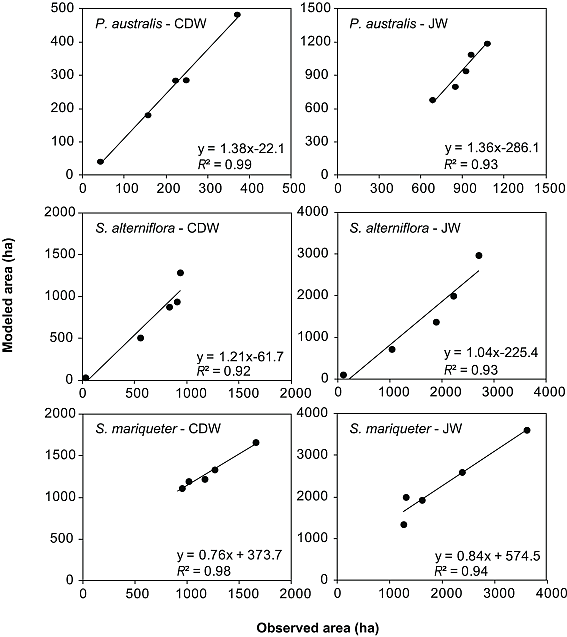


**Figure S2.** Comparison of the spatiotemporal dynamics of the salt marsh vegetation in CDW (left panel) and JW (right panel) between the model calculations and observations during the period of 2000–2011.

**Reference**

1. Zhu, Z. C., Zhang, L. Q., Wang, N., Schwarz, C. & Ysebaert, T. Interactions between range expansion of saltmarsh vegetation and hydrodynamic regimes in the Yangtze Estuary, China. *Estuar. Coast. Shelf Sci.* **96,** 273–279 (2012).
2. Zhang, L. Q. & Yong, X. K. Studies on phenology and spatial pattern of *Scirpus mariqueter* population. *Acta Phytoecol. Geobot. Sin.* **16,** 43–51 (1992)
3. Xiao, D. R., Zhang, L. Q. & Zhu, Z. C. A study on seed characteristics and seed bank of *Spartina alterniflora* at saltmarshes in the Yangtze Estuary, China. *Estuar. Coast. Shelf Sci.* **83,** 105–110 (2009).
4. Li, B. *et al.* *Spartina alterniflora* invasions in the Yangtze River estuary, China: an overview of current status and ecosystem effects. *Ecol. Eng.* **35,** 511–520 (2009).
5. Xiao, D. R., Zhang, L. Q. & Zhu, Z. C. The range expansion patterns of *Spartina alterniflora* on saltmarshes in the Yangtze Estuary, China. *Estuar. Coast. Shelf Sci.* **88,** 99–104 (2010).
6. Huang, H. M. & Zhang, L. Q. A study on the population dynamics of *Spartina alterniflora* at Jiuduansha Shoals Shanghai. *Ecol. Eng.* **29,** 164–172 (2007).
7. Schwarz, C. *et al.* Abiotics governing the establishment and expansion of two contrasting salt marsh species in the Yangtze estuary, China. *Wetlands* **31,** 1011–1021 (2011).
8. Huang, H. M., Zhang, L. Q., Guan, Y. J. & Wang, D.H. A cellular automata model for population expansion of *Spartina alterniflora* at Jiuduansha Shoals, Shanghai, China. *Estuar. Coast. Shelf Sci.* **77,** 47–55 (2008).
9. Chen, Z. Y., Li, B., Zhong, Y. & Chen, J. K. Local competitive effects of introduced *Spartina alterniflora* on *Scirpus mariqueter* at Dongtan of Chongming Island, the Yangtze River estuary and their potential ecological consequences. *Hydrobiologia* **528,** 99–106 (2004).
10. Wang, Q. *et al.* Effects of growing conditions on the growth and interactions between salt marsh plants: implications for invasibility of habitats. *Biol. Invasions* **8,** 1547–1560 (2006).
11. Fagherazzi, S. *et al*. Numerical models of salt marsh evolution: Ecological, geomorphic, and climatic factors. *Rev. Geophys.* **50,** RG1002, doi: 10.1029/2011RG000359 (2012).
12. Li, H. & Yang, S. L. Trapping effect of tidal marsh vegetation on suspended sediment, Yangtze Delta. *J. Coastal Res.* **25,** 915–924 (2009).
13. CWRC (Changjiang Water Resources Commission). Changjiang Sediment Bulletin. Wuhan, Changjiang Press, p. 30–40 (2012).
14. Shi, B., Ma, J. Y. & Wang, K.Y. Effects of atmospheric elevated temperature on the growth, reproduction and biomass allocation of reclamation *Phragmites Australis* in East Beach of Chongming island. *Resour. Environ. Yangtze Basin* **19,** 383–388 (2010).
15. Ge, Z. M., Zhang, L. Q. & Yuan, L. Spatiotemporal dynamics of salt marsh vegetation regulated by plant invasion and abiotic processes in the Yangtze Estuary: observations with a modeling approach. *Estuar. Coast.* **38,** 310–324 (2015).
16. Ge, Z. M., Cao, H. B. & Zhang, L. Q. A process-based grid model for the simulation of range expansion of *Spartina alterniflora* on the coastal saltmarshes in the Yangtze Estuary. *Ecol. Eng.* **58,** 105–112 (2013).
